# Therapeutic Effect of Stem Cells on Male Infertility in a Rat Model: Histological, Molecular, Biochemical, and Functional Study

**DOI:** 10.1155/2021/8450721

**Published:** 2021-10-25

**Authors:** Sally S. Mohammed, Mona F. Mansour, Noha A. Salem

**Affiliations:** ^1^Histology and Cell Biology Department, Faculty of Medicine, Suez Canal University, Egypt; ^2^Medical Physiology Department, Faculty of Medicine, Suez Canal University, Egypt; ^3^Anatomy and Embryology Department, Faculty of Medicine, Suez Canal University, Egypt

## Abstract

Methotrexate (MTX) is a folic acid antagonist, widely used as a chemotherapeutic and immunosuppressive drug, but it is toxic to reproductive systems. In recent years, the era of stem cell applications becomes a promising point as a possible therapeutic agent in male infertility. This study is aimed at evaluating the therapeutic effects of stem cells at histological, molecular, biochemical, and functional levels in a methotrexate-induced testicular damage model. *Material and Methods*. Thirty rats were divided randomly into three groups (ten rats each): group 1 (control): animals received an intraperitoneal injection of 2 ml phosphate-buffered saline per week for 4 weeks, group 2 (MTX-treated group): animals were intraperitoneally injected with methotrexate (8 mg/kg) once weekly for 4 weeks, and group 3 (ADMSC-treated group): methotrexate-treated animals received a single dose of 1 × 10^6^ stem cells/rat at the 5th week. At the 8th week, blood samples were collected for hormonal analysis; then, animals were sacrificed. The testes were dissected; the right testis was stained with hematoxylin and eosin. Random sections were taken from group 3 and examined with a fluorescent microscope. The left testis was divided into two specimens: the first was used for an electron microscope and the second was homogenized for molecular and biochemical assessments. *Results*. Group 2 showed significant histological changes, decreased free testosterone level, decrease in stem cell factor expression, and dysfunction of the oxidation state. The results revealed significant improvement of these parameters. *Conclusion*. Transplantation of adipose tissue-derived stem cells (ADMSCs) can improve the testicular damage histologically and functionally in a rat model.

## 1. Introduction

In recent years, the era of stem cell applications becomes a promising point of research as a possible therapeutic agent in male infertility [1]. Isolation of mesenchymal stem cells from the bone marrow is presented with pain, morbidity, and low cell number. Adipose tissue-derived mesenchymal stem cells (ADMSCs) are derived from the mesenchyme, which contains a supportive stroma that is easily isolated; therefore, it represents a source of stem cells that could have far-reaching effects on several fields [2].

Chemotherapeutic drugs have been reported to cause permanent azoospermia and infertility in men [3]. Methotrexate (MTX) is a folic acid antagonist, widely used as a chemotherapeutic and immunosuppressive drug. Toxic side effects of methotrexate have been demonstrated in various animals. It seems to be hepatotoxic, nephrotoxic, and harmful to reproductive systems [4]. According to different surveys, male factor infertility appeared to be 25%–50% of cases [5]. Testicular damage is the most significant potential side effect of MTX. Studies have reported that MTX induced overproduction of reactive oxygen species (ROS) and oxidative stress. The MTX-induced ROS affect male fertility significantly. ROS causes DNA damage, endothelial dysfunction, and damage to the mitochondrial membrane, with gross injury to seminiferous tubules and apoptosis of spermatogonial stem cells (SSCs) [6].

Bhang et al. showed that testicular endothelial cells (TECs) are part of the SSC niche producing glial cell line-derived neurotrophic factor (GDNF) and other factors to support human and mouse SSCs in long-term culture [7].

ROS also cause damage to the mitochondrial membrane stimulating apoptosis of the testicular germ cell, with subsequent decreased sperm count and sperm DNA damage after its administration [6].

Glutathione (GSH) is an essential cytosolic antioxidant and needs NADPH for its effective action. MTX inhibits the NAD(P)-dependent dehydrogenases and NADP malic enzyme, decreasing the availability of NADPH. Consequently, the effectiveness of GSH as an antioxidant decreases with MTX [8]. Also, it destroys the cell membrane polyunsaturated fatty acids and increases lipid peroxidation and production of malondialdehyde (MDA), which is known as a common oxidative marker [9].

So ADMSCs can repair and regenerate tissues by several mechanisms, and they might secrete cytokines and growth factors that stimulate recovery [10]. Also, it could suppress immune reaction [11]. Spermatogonial stem cells (SSCs) replicate not only for maintaining the stem cell pool but also for differentiating into spermatogonia, spermatocytes, and spermatids. The niche of the testis is the main regulator of fate decisions of SSCs, as it is composed of Sertoli cells (SC), cytokines, growth factors, and blood vessels [12].

Testicular endothelial cells (TECs) represent a key population in the germ cell niche secreting GDNF, fibroblast growth factor-2 (FGF-2), stromal cell-derived factor-1 (SDF-1), macrophage inflammatory protein 2 (MIP-2), and insulin-like growth factor-binding protein 2 (IGFBP-2). ROS-induced dysfunction of testicular endothelial cells disturbs the self-renewal of SSCs [8].

Sertoli cells secrete numerous growth factors such as glial cell line-derived neurotrophic factor (GDNF), stem cell factor (SCF), retinoic acid (RA), and bone morphogenetic protein 4 (BMP4) [13]. GNDF has been demonstrated to stimulate the self-renewal of spermatogonial cells; SCF, BMPs, and RA induce spermatogonial cell differentiation [14]. The SCF is a cytokine that activates the tyrosine kinase receptor c-KIT, which regulates both proliferation and differentiation of spermatogonia and mediates the effects of both RA and BMP4 on spermatogonial cell differentiation [15]. In the present study, we aimed to evaluate the therapeutic role of ADMSCs in methotrexate-induced testicular damage in rat using histological, molecular, molecular, and functional parameters.

## 2. Materials and Methods

### 2.1. Animals and Experimental Design

The study was performed on 30 Sprague-Dawley albino rats weighing between 200 and 250 g purchased from the animal house of the Faculty of Veterinary Medicine, Suez Canal University. All rats were housed under controlled laboratory conditions, and they were fed commercial rat pellets and drinking water, ad libitum. Rats were randomised and divided into three groups (10 per group). Group 1 (control group): animals received an intraperitoneal injection of 2 ml phosphate-buffered saline per week for 4 weeks. Group 2 (MTX-treated group): methotrexate (MTX) is a folic acid antagonist that has antineoplastic characteristics. A methotrexate (Mylan) vial for injection was purchased from Al-Gomhoria Pharmaceutical Company, Cairo, Egypt. Animals were intraperitoneally injected with MTX in a dose of 8 mg/kg body weight once weekly for 4 weeks [16]. Group 3 (ADMSC-treated group): the treated rats with MTX for 4 weeks received a single dose of PKH26-labelled ADMSCs/rat (1 × 10^6^) [17], at the 5^th^ week, via tail vein injection.

The rats were weighed once weekly. At the 8^th^ week, blood samples were collected for hormonal analysis under general anaesthesia. Then, animals were sacrificed through neck dislocation. The testes were gently dissected free from the scrotum and weighed. The right testis was immersed in Bouin's fixation solution, processed into paraffin sections, and stained with haematoxylin and eosin for the light microscopic examination. Random unstained sections were taken from the ADMSC group and examined with immunofluorescent microscopy. The left testis was divided into two specimens: the first one was used for electron microscopic examination and the second specimen was used for homogenization and biochemical analysis.

### 2.2. Preparation of ADMSCs

Retroperitoneal fat tissue was isolated from healthy ten rats aged four weeks, after anaesthesia and scarification. The collected fat was dissected from blood vessels and repeatedly washed with phosphate buffer saline (PBS). Then, it was cut into small pieces of 1 mm × 1 mm and then digested with 0.075% type I collagenase at 37°C for 30 min. High-glucose Dulbecco's modified Eagle's medium containing 10% fetal bovine serum was used to stop the enzyme's reaction; then, centrifugation was done to obtain a precipitate. The cells were seeded in 75 cm^2^ Petri dishes using complete fresh media (Lonza Bioproducts, Belgium, Germany) incubated at 5% CO_2_ for 48 hours. Then, after the third passage, the cells were harvested using 0.25% trypsin for 1 min [18].

### 2.3. Characterization of ADMSCs

The isolated ADMSCs were characterized physically, through the spindle-shaped morphology and their plastic adherence to the culture dishes and immunophenotypically characterized as CD34-ve, CD29+ve, and CD105+ve using flow cytometry monoclonal FITC antibodies (CD34 (catalog # MA1-10204, Invitrogen), CD29 (catalog # 11-0299-42, Invitrogen), and CD105 (catalog # MA1-19594, Invitrogen)). All monoclonal FITC antibodies were assessed according to the manufacturer's instructions.

### 2.4. Labelling of ADMSCs

The characterized CD34-ve, CD29+ve, CD105+ve ADMSCs were labelled with a PKH26 red fluorescent linker dye (Sigma-Aldrich, German, catalogue no. MINI26). Final concentrations of 2 × 10^−6^ M PKH26 dye and 1 × 10^7^ cells/ml in a 2 ml total volume were stained following the manufacturer's instruction.

After harvesting, the cells in the above count were resuspended in 1 ml of diluent C (supplied with the kit), with pipetting to ensure complete dispersion. 1 ml of cell suspension was rapidly added to 1 ml of PKH26 dye, with immediately mixing by pipetting. The sample was incubated at 25°C for 2-5 minutes with gentle inversion of the tube to assure mixing. Then, the reaction was stopped by adding serum for 1 min. The cells were centrifuged at 2000 rpm for 10 minutes at 25°C, and 10 ml of complete culture medium was added to the cells.

### 2.5. Light Microscopic Examination

Three fields per five serial sections from each animal were chosen randomly for histological assessment. The germinal epithelium thickness and the tubular diameter were measured using the Fiji ImageJ program (version 1.52i, National Institutes of Health, Bethesda, MD, USA). The assessment of the germinal epithelium thickness and the tubular diameter was done according to JTBS (Table 1). The examination of the stained sections was carried out by using a Leica DM 1000 light microscope.

### 2.6. Fluorescent Microscopic Examination

Tracking the ADMSCs after animal injections is important to detect their effect on tissues. The PKH26-labelled cells were easily detected through examination of random chosen unstained paraffin sections from the stem cell-treated group, with fluorescence microscopy (Leistungselektronik Jena GmbH, Germany).

### 2.7. Electron Microscopic Examination

The specimens of the left testis were divided into small pieces, immersed for two hours in 2.5%phosphate-buffered glutaraldehyde solution (pH 7. 4) at 4°C, washed with phosphate buffer, and then postfixed for one hour in 1% buffered osmium tetroxide solution. Ultrathin sections were cut using American RMC Ultramicrotome, stained with uranyl acetate and lead citrate, and then examined and photographed with 1200EX *Π* (JEOL, Japan) in an electron microscope unit (Faculty of Science, Ain Shams University, Cairo).

### 2.8. Homogenization of Testicular Tissue

Tissue slices were taken and washed out in 0.01 modified phosphate-buffered saline (MPBS). A tissue protein extraction reagent was added according to the proportion of 1G (5-10 ml) and mixed in ice water. After being blended, the mixture was centrifuged for 10 min at 5000-10000 rpm. Part of the supernatant was tested immediately, and part was stored at -20°C to -80°C.

### 2.9. Molecular Assessment

Western blotting of the testicular tissue homogenate was done to evaluate relative expression of the soluble form of stem cell factor (SCF) in six samples of the frozen testicular tissue homogenate (two from each group). The technique was done according to manufacturer's instructions (Invitrogen, catalog # PA5-79558).

### 2.10. Biochemical Assessment of the Oxidation State

The oxidant-antioxidant status of the testicular homogenate was estimated by measuring the lipid peroxide (malondialdehyde (MDA) (Biodiagnostic, Egypt, cat. no. MD 25 28), reduced glutathione (GSH) (Biodiagnostic, Egypt, cat. no. GR 25 10), and total antioxidant capacity (TAC) (Biodiagnostic, Egypt, cat. no. TA 25 12)). The colorimetric assay of MDA, GSH, and TAC was done according to the manufacturer's instructions (http://www.bio-diagnostic.com).

### 2.11. Functional Assessment of Effective Gametogenesis (Serum-Free Testosterone Level)

Serum-free testosterone level was assessed using the free testosterone ELISA kit (MyBioSource, Egypt (catalog # MBS262755)).

## 3. Statistical Analysis

All data were coded and analysed using the Statistical Package for the Social Sciences (SPSS) software version 19. Results were expressed as means and standard deviations. One-way ANOVA was used to compare the means of different groups. Post hoc tests were done. Correlation and predictive relations between values were done using simple and multivariant regression analysis among two or more variables, respectively. Differences are considered significant when *P* < 0.05.

## 4. Results

### 4.1. Physical Data

Rats' body weight and right and left testis weight were assessed in all study groups. In doing one-way ANOVA, no significant difference was found among different study groups (*P* = 0.2, 0.3, and 0.6, respectively) (data not shown).

### 4.2. Immunophenotypic Characterization of Transplanted ADMSCs

**Figure 1:**
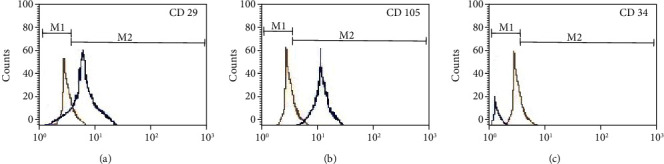
Expression of markers by flow cytometry on transplanted ADMSCs. The ADMSCs showed significant expression of mesenchymal markers (a) CD29 and (b) CD105. (c) The cells showed negative expression for CD34.

After the expansion, the cells obtained from rat retroperitoneal adipose tissue were immunophenotypically characterized by flow cytometry. The cells showed a significant expression of CD29 (79%) and CD105 (70%), as shown in Figures 1(a) and 1(b), respectively, while showing negative expression for CD34 (Figure 1(c)).

### 4.3. Haematoxylin and Eosin Results

**Figure 2:**
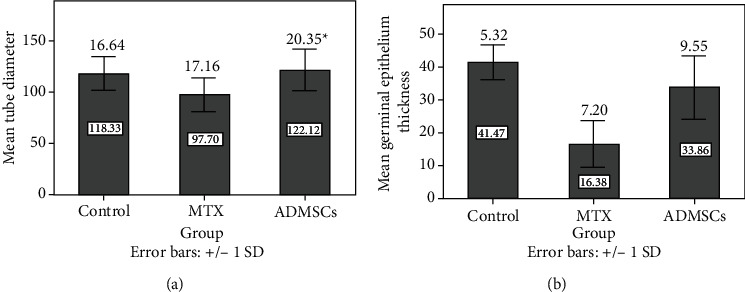
(a) The mean outer diameter of the seminiferous tubules; q indicates a significant difference between MTX and control groups. ∗ indicates a significant difference between MTX- and ADMSC-treated groups. (b) The mean germinal epithelium thickness using the post hoc Tukey test. The mean difference is significant at the 0.05 level, and a statistically significant difference between all groups is present.

**Figure 3:**
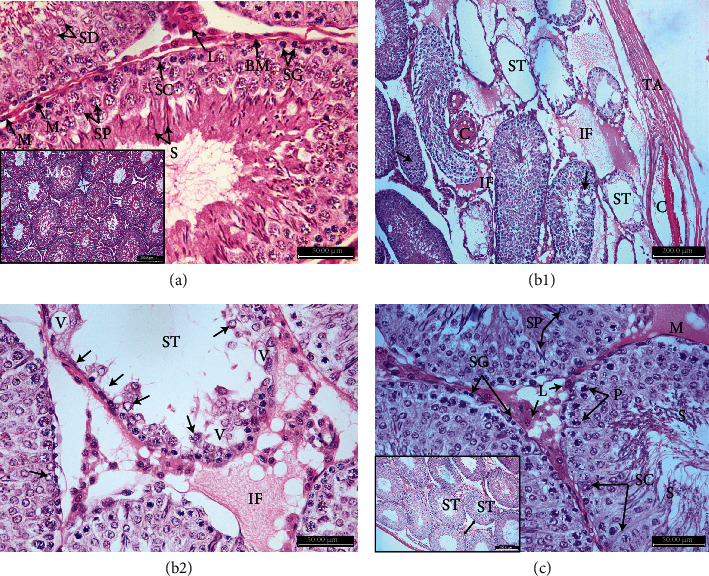
Photomicrographs of H&E-stained sections from the rat testis. (a) Control testis showing normal seminiferous tubules lined by spermatogonia (SG), lying on regular basement membranes (BM), primary spermatocytes (SP), rounded spermatids (SD), mature sperms (S) inside the lumen, Sertoli cells (SC), interstitial tissues containing Leydig cells (L), and flat nuclei of myoid cells (M). (b) MTX group showing disruption of seminiferous tubules (ST) with complete loss of spermatogenic cells and Sertoli cells and some tubules with sloughing of germ cells into the tubular lumens with loss of sperms (arrowheads). Congested blood vessels (C), tunica albuginea (TA), and oedema in interstitial tissue (IF). (c) MTX group showing complete loss of spermatogenic cell lines and Sertoli cells (arrows), cells having nuclei with chromatin margination (arrowheads), vacuolations (V) with loss of mature sperms, and oedema in interstitial tissue (IF). (d) ADMSC-treated testis showing normal seminiferous tubules and myoid cells (M), lined by normal spermatogonia (SG), spermatocytes (P), spermatids (SP), sperms (S), and triangular Sertoli cell (SC) resting on the intact basement membrane. Interstitial tissues contain Leydig cells (L) (H&E ×400, photo inside frame, ×100).

The histological pattern of the rat testis in the control group showed normal architecture of seminiferous tubules with a normal rounded or oval outline. It was surrounded by regular basement membranes and myoid cells. The tubules were lined with the normal germinal epithelium. More than 80% of the examined tubules had a score of ten (Table 2, Figure 2). Spermatogonia were small dome-shaped, with vesicular or opaque nuclei, large polygonal primary spermatocytes. Small rounded early spermatids on the top of cells and late elongated spermatids attached to the apex of Sertoli cells with a small amount of cytoplasm and small condensed nuclei; meanwhile, the tails of mature liberated sperms were present inside the tubule's lumen. Sertoli cells appeared as triangular cells lying on basement membranes. Interstitial tissues contained blood vessels, clusters of polygonal acidophilic cells with rounded central open-face nuclei, and Leydig cells. The tubules were surrounded by myoid cells with flat nuclei (Figure 3(a)).

Sections from the MTX group revealed severe disruption of seminiferous tubules with an irregular outline and significant shrinkage of their diameters and a decrease in the germinal epithelial thickness (Figure 2). With complete loss of spermatogenic cell lines and Sertoli cells, some seminiferous tubules showed sloughing of germ cells into the tubular lumen; others had vacuolations and loss of mature sperms, with a significant decrease in the values of the Johnson score as shown in Table 2. Congested blood vessels in tunica albuginea and tunica vasculosa with interstitial oedema were observed (Figures 3(b) and 3(c)).

Sections from ADMSC-treated rats yielded a reversal of the histopathological changes found in MTX-treated rats. The tubule outline was regular with little interstitial connective tissue in between, which contained groups of Leydig cells, similar to the control group. There was restoration of the normal germinal and Sertoli cells lining the tubules with a significant increase in the seminiferous tubule's diameter and germinal epithelium thickness. There was also a significant restoration of the Johnson score, as more than 40% of the examined tubules had a score of 9. These results were comparable to those of the control group (Table 2, Figures 3(d) and 2).

### 4.4. Fluorescent Microscopy

**Figure 4:**
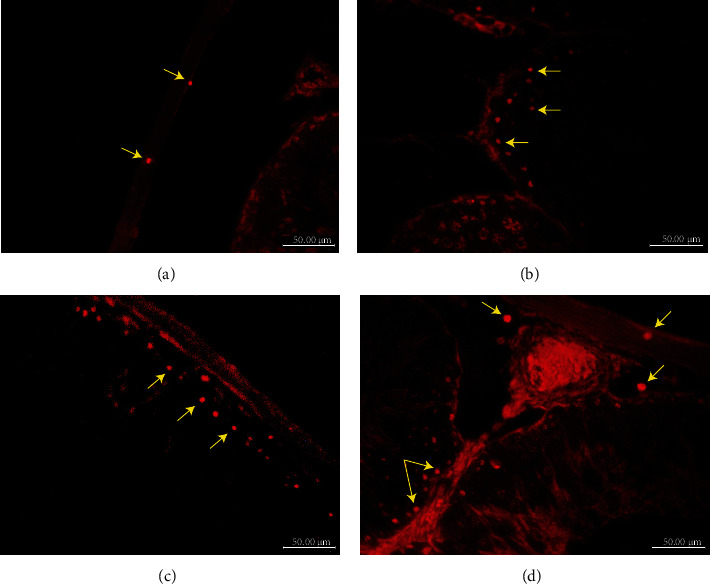
Immunofluorescent photos show the fluorophore of the labelled stem cells in testicular tissue of the ADMSC-treated group. (a, b) Transplanted stem cells in the tunica albuginea (arrows). (c) Fluorophores lining the seminiferous tubules (d) in interstitial tissue (arrows).

As the injected MSCs were labelled with a fluorescent dye, detecting homing of the cells inside the testis tissue was done efficiently. Imaging the unstained testicular sections yielded the red fluorophore of the labelled cells in the tunica albuginea, interstitial tissue, and lining of the seminiferous tubules (Figure 4).

### 4.5. Transmission Electron Microscopic Results

#### 4.5.1. Control Group

**Figure 5:**
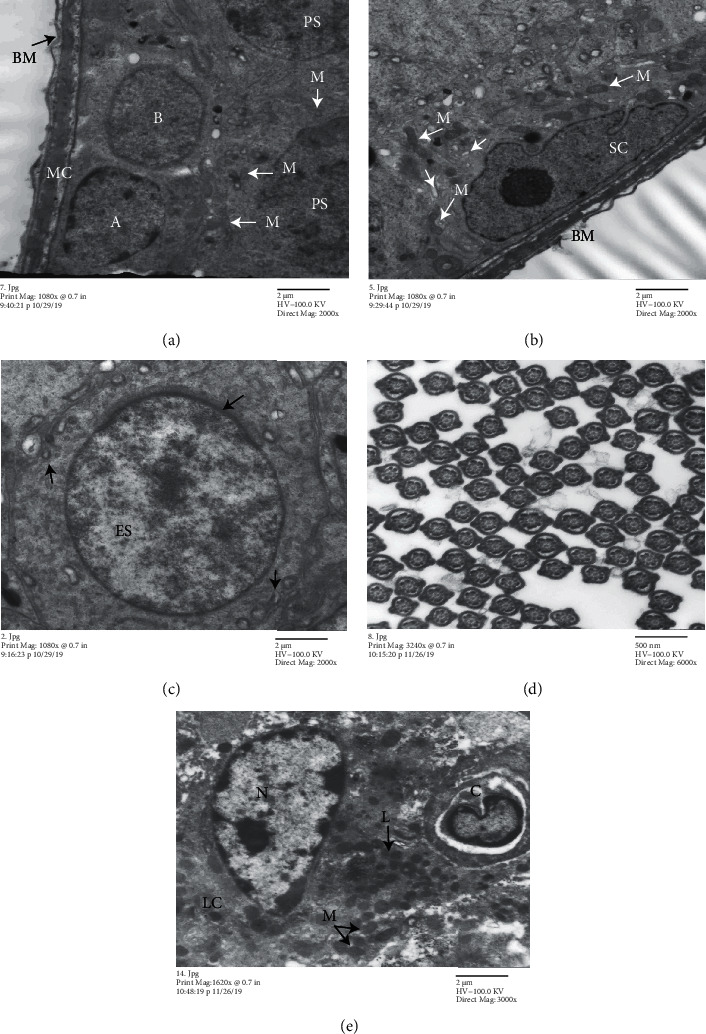
Electron micrographs of a seminiferous tubule of a control rat. (a) Normal basement membrane (BM), the myoid cell with the flat nucleus (MC), mitochondria (M), type A and B spermatogonia, and primary spermatocyte (PS). (b) Sertoli cell (SC), mitochondria (M), and smooth endoplasmic reticulum (sER) (arrowhead). (c) Early spermatid (ES), apical acrosomal cap (arrow), and peripheral mitochondria (arrowhead). (d) Cross section in mature sperms inside the tubule lumen having a central axoneme surrounded by outer dense fibres and fibrous sheath. (e) Interstitial tissue with blood capillary (C), Leydig cells (LC), large open-face nucleus (N), mitochondria (M), and lipid droplets (L) ((a)–(c): TEM, ×2000; (d): ×6000; and (e): ×3000).

Normal testicular ultrastructure was observed. The seminiferous tubules had regular basement membranes containing normal myoid cells with flat nuclei, lined by two types of spermatogonia: types A and B. Type A spermatogonia were characterized by large pale ovoid nuclei containing euchromatin and peripheral chromatin. Type B spermatogonia were smaller and contained rounded nuclei with more euchromatin. Primary spermatocytes had a large spherical nucleus with faint granular chromatin and spherical mitochondria peripherally (Figure 5(a)). Sertoli cells extending radially from the basement membrane to the lumen of the tubule had a large pale nucleus, many smooth endoplasmic reticula, and numerous mitochondria (Figure 5(b)). Early spermatid was rounded and large and had peripheral clusters of mitochondria and a large spherical euchromatic nucleus covered with an apical acrosomal cap (Figure 5(c)). Moreover, the lumen contained many transverse sections of normal sperms (Figure 5(d)). Normal interstitial tissue contains Leydig cells near blood capillaries. The cells had a large euchromatic nucleus with peripheral chromatin and prominent nucleolus and numerous mitochondria and lipid droplets (Figure 5(e)).

#### 4.5.2. Methotrexate-Treated Group

**Figure 6:**
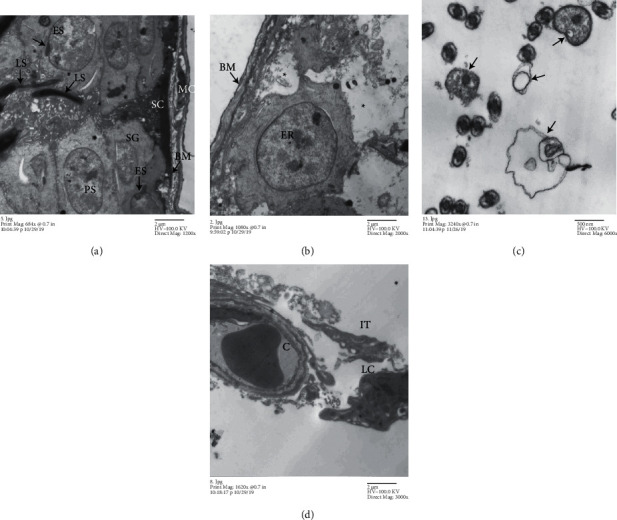
Electron micrographs of a seminiferous tubule of the MTX-treated group. (a) Distracted basement membrane (BM) with damaged myoid cells (MC), spermatogonia (SG) separated from the basement membrane, damaged Sertoli cells (SC) with a dark, shrunken nucleus, primary spermatocyte (PS), early spermatids (ES) with an abnormal acrosomal cap (arrowhead), and late spermatids (LS). (b) Necrosis and disappearance of certain stages of germ cells with cellular debris, widening of intercellular spaces in between (∗) and loss of Sertoli cells, and early spermatid (ER) with an acrosomal cap. (c) Transverse sections of distorted and malformed sperms in the lumen of the seminiferous tubules (arrows). (d) Abnormal wide interstitial tissue (IT) with degenerated Leydig cell (LC) and blood capillary(C) ((a): TEM, ×1200; (b): ×2000, (c): ×6000; and (d): 3000).

Loss of the normal architecture and distraction of the basement membranes with distorted myoid cells were observed. We observe damage of spermatogonia and apoptosis of Sertoli cells with severely shrunken dark nuclei and little dark cytoplasm. Primary spermatocytes were necrotic and lost or had an abnormal nucleus (Figures 6(a) and 6(b)). Early and late spermatids appeared in the wrong place at the basal compartment. Abnormal acrosomal capping appeared in some early spermatids with malformed sperms (Figures 6(a) and 6(c)). There is abnormal widening of interstitial tissue with degenerated Leydig cells containing an irregular shrunken nucleus with clumped heterochromatin (Figure 6(d)).

#### 4.5.3. ADMSC-Treated Group

**Figure 7:**
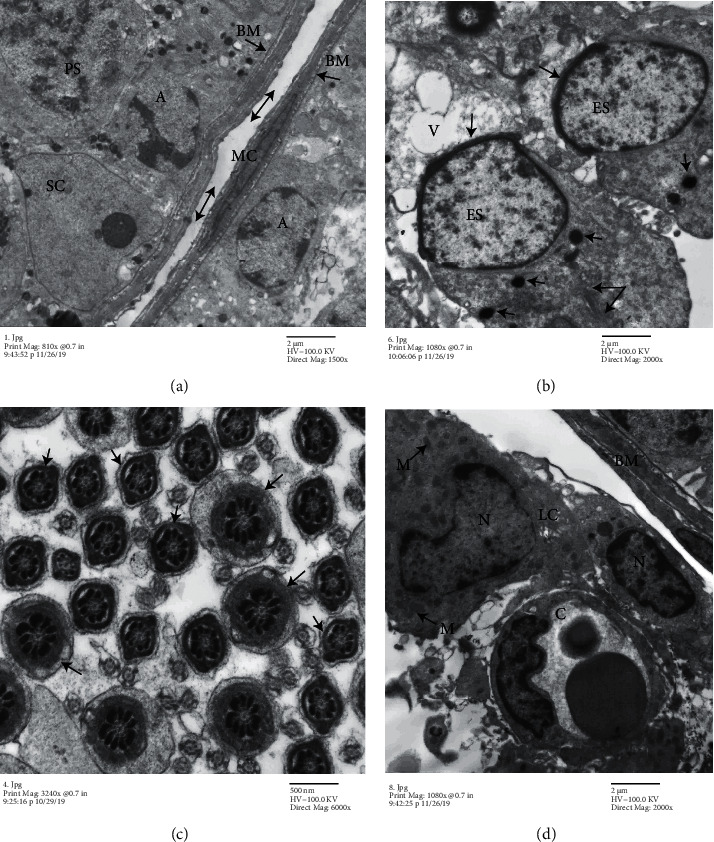
Electron micrographs of a seminiferous tubule of the ADMSC-treated group. (a) Regular basement membrane (BM) with normal myoid cells (MC). Normal space between two seminiferous tubules (double head arrow). Normal type A spermatogonia (A), primary spermatocyte (PS), and Sertoli cell (SC). (b) Early spermatid (ES) with an acrosomal cap (arrow), lipid droplets (arrowhead), and mitochondria around a growing axoneme (double arrow). Few vacuolations in between the cells (V). (c) The lumen of a seminiferous tubule contains different transverse sections of normal sperms and central axoneme with mitochondrial sheath (arrow) and fibrous sheath (arrowhead). (d) Interstitial tissue contains Leydig cells (LC) with euchromatic nuclei (N), mitochondria (M), and blood capillary (C) ((a): TEM, ×1500; (b, d): ×2000; and (c): ×6000).

The ultrastructure of testicular tissues showed restoration of normal tissues with a regular basement membrane and normal myoid cell. The tubules were lined with normal spermatogonia, primary spermatocytes had large euchromatic nuclei, and Sertoli cells appeared with large euchromatic nuclei (Figure 7(a)). Few spaces of vacuolations still present between germ cells and elongating early spermatids that had normal capping. The sperm sections showed different stages: central axoneme surrounded by outer dense fibres and mitochondrial sheath, fibrous sheath, or plasmalemma (Figures 7(b) and 7(c)). The interstitial tissue was comparable to that of the control group (Figure 7(d)).

## 5. Molecular and Functional Assessments

### 5.1. Assessment of Gametogenesis by the Western Blot Assay of the Stem Cell Factor (SCF) in Homogenized Testicular Tissue

**Figure 8:**
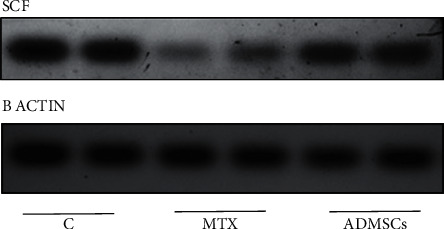
Western blot assay of SCF in homogenized testicular tissue.

**Figure 9:**
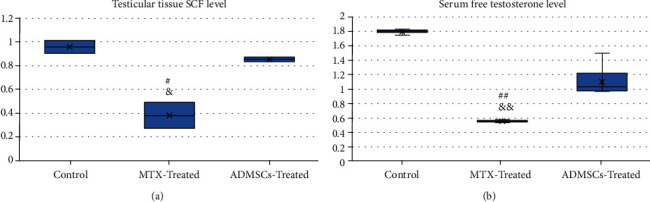
Box blot graph of (a) testicular tissue SCF level (relative expression) and (b) serum testosterone level (ng/ml) among different study groups. All data were expressed as means ± SD and were analysed using one-way ANOVA and Bonferroni post hoc test. ^#^*P* < 0.05 (significant), ^##^*P* < 0.001 (highly significant) compared to the control group; ^&^*P* < 0.05 (significant), ^&&^*P* < 0.001 (highly significant) compared to the MSC-treated group.

The western blot assay of SCF relative expression was done in six samples of homogenized testicular tissue, two samples from each group (Figure 8). As shown in Figure 9(a), a significant difference in testicular SCF expression level was detected among study groups (*P* = 0.021). SCF expression significantly decreased in the MTX-treated group compared to the control group (*P* = 0.022); however, ADMSC transplantation significantly increases the SCF expression compared to the MTX-treated group (*P* = 0.038).

### 5.2. Assessment of the Male Sex Hormone (Free Testosterone) Level in Serum

Serum level of free testosterone level was assessed using ELISA. A highly significant difference was detected among all study groups (*P* < 0.001), with a highly significant decrease in serum testosterone level in both MTX- and ADMSC-treated groups compared to the control group (*P* < 0.001)(Figure 9 (a)). However, the ADMSC group showed a highly significant improvement in its serum testosterone level compared to the MTX-treated group (*P* < 0.001) (Figure 9(b)).

### 5.3. Justification of Testicular Tissue SCF and Serum Testosterone Level Relation

**Figure 10:**
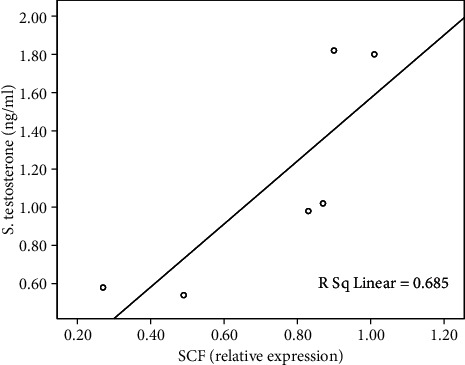
Scatter blot of serum level of free testosterone and SCF in homogenized testicular tissue. The simple regression model was significant, *F* (1‐4) = 8.685, *P* < 0.04, and *R*^2^ = 0.685.

The functional assessment of effective gametogenesis, represented in serum testosterone level, showed a strong positive significant correlation with the testicular level of SCF (*r* = 0.827), and the regression model was significant, *F* (1‐4) = 8.685, *P* < 0.04, and *R*^2^ = 0.685, indicating that testicular SCF expression accounts for 68.5% of the variance in serum testosterone level (Figure 10).

### 5.4. Assessment of Oxidant-Antioxidant Status in Homogenized Testicular Tissue

**Figure 11:**
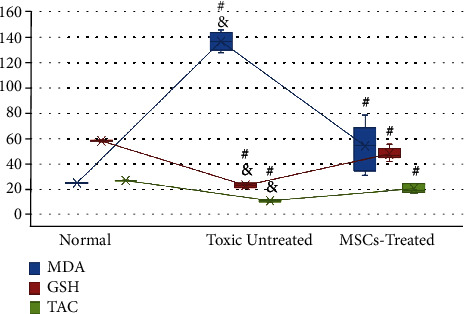
Box blot graph showing means of testicular MDA, GSH, and TAC levels in different study groups. All data were expressed as means ± SD and were analysed using one-way ANOVA and Bonferroni post hoc test. # indicates a highly significant difference compared to the normal group (*P* < 0.001). & indicates a highly significant difference compared to the MSC-treated group (*P* < 0.001).

Testicular level of TAC, GSH, and MDA was assessed to determine the possible role of oxidant-antioxidant imbalance in MTX-induced testicular injury. A highly significant difference was detected among study groups (*P* < 0.001). The antioxidant capacity, represented in TAC and GSH, showed a highly significant decrease in both the MTX-treated group and ADMSC group compared to the control group (*P* < 0.001). Also, lipid peroxidation, represented in MDA, was significantly higher in both MTX- and ADMSC-treated groups compared to the control group (*P* < 0.001). However, the ADMSC group showed a significant improvement in its oxidant-antioxidant imbalance, compared to the MTX-treated group, with highly significant improvement in its TAC and GSH levels (*P* < 0.001) and a highly significant decrease in its MDA level compared to the MTX-treated group (*P* < 0.001) (Figure 11).

### 5.5. Justifying the Role of Oxidative Stress in the Study Plane

#### 5.5.1. Correlation between Oxidative Stress and Testicular SCF Expression

Using the Spearman correlation, testicular SCF showed a significant positive correlation with testicular TAC and GSH (*r* = 0.89 and 0.87, respectively), while a significant negative correlation with MDA (*r* = −0.93) was found. The overall regression model was significant; in doing multiple regression analysis, *F* (3‐26) = 100.76, *P* < 0.001, and *R*^2^ = 0.92, indicating that testicular levels of TAC, MDA, and GSH account for 92% of the variance in testicular SCF expression.

#### 5.5.2. Correlation between Oxidative Stress and Serum Level of Free Testosterone

**Figure 12:**
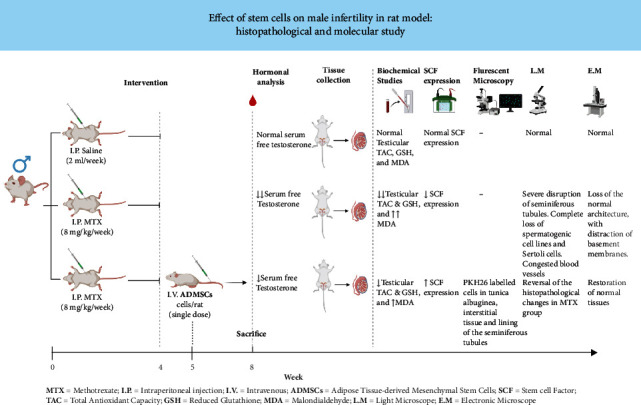
Summary of the used experimental methods and results.

Using the Pearson correlation, serum testosterone showed a significant positive correlation with testicular TAC and GSH (*r* = 0.91 and 0.92, respectively), while a significant negative correlation with MDA (*r* = −0.92) was found. In doing multiple regression analysis, the overall regression model was significant, *F* (3‐26) = 63.09, *P* < 0.001, and *R*^2^ = 0.879, indicating that testicular levels of TAC, MDA, and GSH account for 87.9% of the variance in serum testosterone level. The summary of the used experimental methods and results is shown in Figure 12).

## 6. Discussion

This study was designed to clarify the curative role of adipose tissue-derived mesenchymal stem cells against the effects of methotrexate. It is widely used in the treatment of cancers and autoimmune diseases despite its undesirable effects, especially on the testis and male fertility. In studying the effect of MTX on the rat body and testis weight (right and left), there was no significant difference compared to the control rats [4, 20]; we also found no significant difference.

The H&E-stained testicular sections revealed congestion with interstitial oedema, damage and loss of the germinal cells and Sertoli cells, Leydig cell degeneration, decreased mean germinal epithelium thickness, and tubule diameter indicating decreased spermatogenesis and infertility. These histopathological changes come in agreement with previous studies which reported that MTX induces degeneration and atrophy of seminiferous tubules, sloughing of germ cells away from the basal lamina, decrease in quantity and quality of sperms, and severe apoptosis in the germinal epithelium [19, 21, 22]. Damage to the germinal epithelium resulted in a significant decrease in the Johnson score; the same was found by [21, 23].

The electron microscopic results showed severe damage to the germinal cells, Sertoli cells, and Leydig cells, confirming the results of the light microscope. Our results were in agreement with [24]. They investigated the protective effect of proanthocyanidin against the MTX-induced testicular damage and found distortion of the germinal cells with separation in between apoptotic Sertoli and fragmented Leydig cells.

Stem cell factor (SCF) secreted from Sertoli cells plays an important role in inducing spermatogonial cell differentiation [14]. In our study, MTX toxicity significantly decreased the SCF factor expression from the Sertoli cells in comparison to the control group, resulting from severe damage to the cell evidenced by the light and electron microscopic results. The present results showed a significant decrease in serum-free testosterone level due to the injury that occurred in Leydig cells following the MTX administration, which was in accordance with [6, 25]. Testosterone has an important role in spermatogenesis because it stimulates protein synthesis in all types of spermatogenic cells, leading to sperm development. So, the decrease in testosterone secretion causes impairment of protein synthesis in germ cells with subsequent degenerative changes of germ cells [26].

The possible underlying mechanism mediating MTX-induced testicular damage was sought. It induces oxidative stress damage to testicular tissue through the oxidative-antioxidant imbalance that was evidenced in the current study. MTX administration caused a significant increase in lipid peroxidation (increased MDA) and significant deterioration of antioxidant capacity (decreased GSH and TAC) compared to the control group. This antioxidant imbalance leads to increased free radical production, DNA damage, cellular apoptosis, and arrest of normal spermatogenesis. In the current study, the degree of oxidant-antioxidant imbalance was strongly correlated with the degree of testicular damage represented in a decrease in SCF expression from Sertoli cells and serum-free testosterone level. Similar results were reported by [21, 27, 28].

In recent years, the era of stem cell applications becomes a promising point of research as a possible therapeutic agent in male infertility [1]. Adipose tissue-derived mesenchymal stem cells represented multipotent adult stem cells because of their relative abundance and ease of isolation [29] with high proliferative capacity and production of a huge cellular number and can be expanded for longer periods of time than bone marrow-derived stem cells [30].

In the present study, the ADMSC-treated group showed improvement in the structure and function of testicular tissue. The H&E-stained testicular sections revealed normal lining of the seminiferous tubules which were separated with normal interstitial tissue that contained normal Leydig cells with significant improvement of the Johnson score compared to that of the MTX-treated group. The electron microscopic results confirmed the light microscopic ones. These results were explained by the therapeutic effect of the transplanted ADMSCs after their homing inside the testicular tissue. That homing was detected through the immunofluorescent examination of the testicular tissues from this group. Also, there was a significant increase in the SCF level in the homogenized tissue compared to that of the MTX group, due to restoration of the normal structure and function of Sertoli cells after stem cell transplantation with an increase in the free testosterone level, compared to the MTX-treated group, due to restoration of the normal structure and function of Leydig cells.

In line with our results, Hsiao et al. [31] found that mesenchymal stem cells from adipose tissue protect germ cells from testicular torsion-induced infertility through reducing oxidative stress, preventing apoptosis, and supporting spermatogenesis with the secretion of SCF after local injection of MSCs. Ommurugan et al. [32] concluded that mesenchymal stem cells derived from the bone marrow of humans can be an effective therapy of MTX-induced gonadotoxicity in rats and thus can contribute to the treatment of infertility.

ADMSCs could stimulate spermatogenic cells to proliferate and complete their division to restore normal spermatogenesis [33]. Also, ADMSCs secrete a group of growth factors, such as vascular endothelial cell growth factor, which reduces apoptosis, insulin-like growth factor-1, which is believed to stimulate stem cell proliferation, and hepatocyte growth factor, which acts as an antiapoptotic factor. These secreted cytokines could induce mRNA and protein expression to repair the damaged testes [34]. The potentiating effect of ADMSCs on spermatogenesis was evidenced by a significant increase in SCF expression from the Sertoli cells and serum-free testosterone level in the ADMSC group compared to the MTX group. These results were in agreement with Karimaghai et al. [35] who evaluated the histomorphometric effect of ADMSC allotransplantation on regeneration of germinal cells of seminiferous tubules in busulfan-induced azoospermic hamsters.

So one of the important results of our study in clinical usage is that ADMSCs could be a beneficial treatment for treating male infertility resulting from methotrexate administration. Male patients on chemotherapy may cryopreserve adipose tissue MSCs before starting chemotherapy to be further used as supportive therapy after finishing the chemotherapy schedule.

Studying mature sperm morphology and motility is one of the limitations of our study, as well as the detection of the effect of MTX on the epididymis.

## 7. Conclusion

Adipose-derived mesenchymal stem cell (ADMSC) transplantation alleviated the oxidative stress-induced testicular injury, improving Sertoli cell damage. This, in turn, restores the normal testis niche, stimulating spermatogonia cell proliferation and differentiation. So it can improve the testicular damage histologically and functionally in a rat model. Thus, methotrexate should be used judiciously as it affects the testes, causing a decrease in the fertility rate, so it is safer to use this drug in patients who have completed their family. Studying the incidence of pregnancy after mating and following the embryogenesis is one of our suggestions for future studies in this area.

## Figures and Tables

**Table 1:** Evaluation of the right testicular tissue using the Johnson tubular biopsy score (JTBS) for grading the damage [36].

Score 10	Complete spermatogenesis with many spermatozoa
Score 9	Disorganized spermatogenesis with many spermatozoa
Score 8	Disorganized spermatogenesis with few spermatozoa
Score 7	No spermatozoa but many spermatids
Score 6	No spermatozoa and only a few spermatids
Score 5	No spermatozoa or spermatids and many spermatocytes
Score 4	Few spermatocytes
Score 3	Only spermatogonia
Score 2	Only Sertoli cells
Score 1	No cells

**Table 2:** Johnson scores among the three groups.

Groups	10	9	8	7	6	5	4	3	2	1
Control	84.8	8.9	6.3	0	0	0	0	0	0	0
MTX	3^q^	9	10.3	14^q^	0	12.7^q^	5.1^q^	31.9^q^	0	14^q^
ADMSCs	16.5^∗^	40.3^∗^	7.6	22.1	0	6.3	0	5.1^∗^	0	2.1^∗^

^q^A significant difference between MTX and control. ^∗^A significant difference between ADMSCs and MTX. The difference is considered statistically significant at the 0.05 level, using the chi-square test.

## Data Availability

Data are available on reasonable request.
